# EEG spectral power in developmental coordination disorder and attention-deficit/hyperactivity disorder: a pilot study

**DOI:** 10.3389/fpsyg.2024.1330385

**Published:** 2024-05-03

**Authors:** Emily J. Meachon, Marlene Kundlacz, Kate Wilmut, Georg W. Alpers

**Affiliations:** ^1^School of Social Sciences, University of Mannheim, Mannheim, Germany; ^2^Faculty of Psychology, University of Basel, Basel, Switzerland; ^3^Centre for Psychological Research, Faculty of Health and Life Sciences, Oxford Brookes University, Oxford, United Kingdom

**Keywords:** electroencephalography, neurodevelopmental disorders, dyspraxia, oscillations, resting state

## Abstract

Developmental coordination disorder (DCD) and attention-deficit/hyperactivity disorder (ADHD) overlap in symptoms and often co-occur. Differentiation of DCD and ADHD is crucial for a better understanding of the conditions and targeted support. Measuring electrical brain activity with EEG may help to discern and better understand the conditions given that it can objectively capture changes and potential differences in brain activity related to externally measurable symptoms beneficial for targeted interventions. Therefore, a pilot study was conducted to exploratorily examine neurophysiological differences between adults with DCD and/or ADHD at rest. A total of *N* = 46 adults with DCD (*n* = 12), ADHD (*n* = 9), both DCD + ADHD (*n* = 8), or typical development (*n* = 17) completed 2 min of rest with eyes-closed and eyes-open while their EEG was recorded. Spectral power was calculated for frequency bands: delta (0.5–3 Hz), theta (3.5–7 Hz), alpha (7.5–12.5 Hz), beta (13–25 Hz), mu (8–13 Hz), gamma (low: 30–40 Hz; high: 40–50 Hz). Within-participants, spectral power in a majority of waveforms significantly increased from eyes-open to eyes-closed conditions. Groups differed significantly in occipital beta power during the eyes-open condition, driven by the DCD versus typically developing group comparison. However, other group comparisons reached only marginal significance, including whole brain alpha and mu power with eyes-open, and frontal beta and occipital high gamma power during eyes-closed. While no strong markers could be determined to differentiate DCD versus ADHD, we theorize that several patterns in beta activity were indicative of potential motor maintenance differences in DCD at rest. Therefore, larger studies comparing EEG spectral power may be useful to identify neurological mechanisms of DCD and continued differentiation of DCD and ADHD.

## 1 Introduction

Developmental Coordination Disorder (DCD) and Attention-Deficit/Hyperactivity Disorder (ADHD) are common neurodevelopmental disorders, each affecting about 5% of the population (Thomas et al., 2015; Blank et al., 2019). Despite their unique diagnostic specifications in the DSM-5, DCD and ADHD overlap in many secondary symptoms, including motor and executive functioning difficulties, and can be challenging to disentangle (American Psychiatric Association, 2013; Kaiser et al., 2015; Meachon et al., 2022). In addition, DCD and ADHD co-occur in about 50% of cases and it is not clear if co-occurrence is driving the symptom overlaps or vice versa (Blank et al., 2019). In previous studies, surmounting evidence shows that adults in particular do not have significantly different objective task performance when their symptoms are engaged, but can differ in underlying mechanisms observed at the neural level (e.g., via inhibition: MacLaren et al., 2007; Meachon et al., 2021). Therefore, it is important to differentiate DCD and ADHD by examining neural mechanisms of one or both conditions.

Some studies have shown there are functional differences in the brain between children with DCD and/or ADHD during cognitive tasks (e.g., McLeod et al., 2014) and at rest (e.g., McLeod et al., 2016; Rohr et al., 2021). Furthermore, neural differences are often particular to individuals with co-occurring DCD and ADHD as opposed to just one condition or those of typical development (McLeod et al., 2014; Langevin et al., 2015; Thornton et al., 2018). However, the detection of differences in neural activity between DCD and ADHD has been mixed: some studies found key distinctions (McLeod et al., 2014, 2016) and others noted subtle to no differences (Thornton et al., 2018; Rinat et al., 2020). In addition, it is still unclear if individuals with DCD and/or ADHD have different baseline activity in the resting state and, to our knowledge, this has not been explored among adult groups (Tallet and Wilson, 2020; Meachon et al., 2021). Therefore, the present study exploratorily investigated oscillatory resting state electrical brain activity (i.e., alpha, beta, theta, delta, gamma, and mu) via electroencephalography (EEG) in adults with DCD and/or ADHD.

We first provide an overview of resting state measurement with EEG, describe the existing evidence of resting state activity in DCD or ADHD, and detail the known symptomatic overlaps of DCD and ADHD. While the resting state is often equated to measurement of the default mode network in imaging and connectivity research (Mak et al., 2017), for clarity, we use the term “rest state” throughout the paper to describe EEG measurement not explicitly intended to assess connectivity.

### 1.1 Resting state measurement with EEG

EEG is used to measure the electrical impulses of axonal activity in near-surface regions of the brain (Teplan, 2002). Numerous experiments have used EEG to gain insights into the underlying neural activity in relation to various disorders in biological and psychological science (e.g., Michel et al., 1993; Luck, 2014). EEG is considered an essential tool in several research fields (e.g., attention and speech development), in diagnosis (e.g., epilepsy and sleep disorders; O’Sullivan et al., 2006), and it provides highly accurate temporal resolution not achievable with other neurophysiological measures (e.g., NIRS and MRI).

EEG is often used to capture spectral power through specific frequencies of electrical activity, also known as oscillations. Oscillatory activity can be used to infer general information about one’s conscious state and can be used to discriminate various disorders of consciousness and to predict several cognitive functions such as attention and fluid intelligence (White and Siegel, 2016; Corchs et al., 2019; Rogala et al., 2020). There are four major forms of wavelengths observed in EEG, including: alpha, beta, theta, and delta which occur within unique ranges (in Hertz: Hz) and have different associations. The alpha band has a range of around 8–12 Hz and signifies a relaxed state characterized by medium to large amplitudes (10–150 microV; Klimesch, 1999) theorized to reflect underlying inhibition and cognition relevant to attention (Klimesch, 2012). Beta frequencies occur in a range around 14–25 Hz and indicate focused wakefulness, characterized by small amplitudes (<25 microV; Lubar et al., 1995) which may reflect the presence or absence of maintaining one’s cognitive or sensorimotor state (Engel and Fries, 2010). The theta band is often considered a marker of attentional control and has a range of about 4–7 Hz with large amplitudes (>50microV; Cavanagh and Frank, 2014). The range of delta waves and their implications are generally more variable than other frequency bands, but occur during slow-wave sleep (Dijk et al., 1990) and may interfere with one’s ability to complete a cognitive task (Harmony, 2013).

An even more ambiguous frequency is the gamma band which occurs at frequencies of 25 Hz or greater in wakeful and sleep states (Mably and Colgin, 2018). Gamma activity is thought to be non-specific in function but can occur in response to sensory stimuli and higher order cognitive processing (Başar, 2013; Mably and Colgin, 2018). Gamma frequency bands were among the least reported frequencies in studies examining resting state electrophysiology in psychiatric conditions (Newson and Thiagarajan, 2019). Finally, the mu frequency which occurs around 8–13 Hz, is thought to reflect cognitive processing when paired with beta bands and motor processing, motor imagery, perception, and/or action in combination with alpha bands (Pineda, 2005; Pfurtscheller et al., 2006; Démas et al., 2020). While the higher end of the mu frequency spectrum is thought to be specific to central region and motor-related activity, the higher end of the spectrum is considered to be generalized in both aspects (Thorpe et al., 2016). Some have suggested mu is specific to sensorimotor regions whereas the overlapping alpha frequency is more relevant to the occipital cortex, they are highly difficult to distinguish (e.g., Garakh et al., 2020). For example, when measured with in adults EEG alone, mu rhythms can distribute more widely and should be considered across the cortical surface (e.g., Thorpe et al., 2016).

In typical resting state EEG measurement, participants sit still with open or closed eyes for several seconds to minutes at a time. Rest state activity can differ substantially between conditions with eyes-open and eyes-closed, especially because when participants’ eyes are open, they are naturally exposed to more visual stimuli associated with arousal levels, e.g., via skin conductance (Barry et al., 2007, 2009; Alba et al., 2016). In the eyes-open resting condition, amplitudes in all four major frequency bands are typically reduced compared when one’s eyes are closed and the alpha band in particular is highly relevant to the resting state because it primarily indexes resting state-related arousal rather than activation indicative of visual processing changes from eyes-closed to eyes-open conditions (e.g., Barry et al., 2007; Barry and De Blasio, 2017).

### 1.2 Resting state brain activity in DCD and ADHD

Several studies have examined differences in resting state neurophysiological activity in children with DCD alone compared to typically developing children (De Castelnau et al., 2008), or ADHD alone compared to typically developing children (e.g., Liechti et al., 2013; Buyck and Wiersema, 2014; Alba et al., 2016). However, the neurophysiological evidence surrounding DCD in the resting state is particularly limited. It has been theorized that symptoms of DCD are related to a difference in frequency band coherence in the brain during motor tasks (Tallet and Wilson, 2020). Accordingly, DCD has been dubbed a “disconnection syndrome” due to alterations in connectivity between different areas of the brain among individuals with DCD (Tallet and Wilson, 2020). One EEG study examined spectral coherence during different motor tasks among children with DCD, with motor tasks varying from simple to difficult (De Castelnau et al., 2008). The alpha and beta frequency bands showed increased coherence in children with DCD compared to typically developing controls, which was likely related to sensorimotor activation (De Castelnau et al., 2008). In this case, higher coherence is considered to be more dysfunctional and increased with heightened task difficulty (De Castelnau et al., 2008). These results provided evidence that children with DCD have a higher cognitive load while performing motor tasks and reduced connectivity during these tasks. Furthermore, Keating et al. (2023) recently reported that children with DCD showed reduced synchronization of mu oscillations during movement and reduced mu power while observing a moving kaleidoscope pattern compared to typically developing children. However, no differences in mu and alpha activity were detected at rest between groups or between eyes-open and eyes-closed trials (Keating et al., 2023). While this suggests a role of mu in movement relevant to DCD, it is unclear why activity did not differ between eyes-open and eyes-closed trials.

Contrary to the limited evidence for DCD, the resting state has been examined in a plethora of EEG studies about ADHD (e.g., Barry et al., 2011; González et al., 2013; Buyck and Wiersema, 2014; Alba et al., 2016; Rommel et al., 2017; Clarke et al., 2020). It is considered a robust finding that absolute delta power is increased in individuals with ADHD during eyes-closed compared to typically developing individuals (Newson and Thiagarajan, 2019). The theta-beta ratio has also been suggested as a biomarker for ADHD, however, a review of 65 studies of the resting state in ADHD showed this result is inconsistent in adults and likely dependent on age (Newson and Thiagarajan, 2019). This was confirmed in several studies, such as Kiiski et al. (2022), who identified numerous features of absolute and relative spectral power relevant to predicting ADHD in adults which did not include the theta/beta ratio. For example, increased power in delta and theta spectral power, could successfully classify those with ADHD from typically developing individuals (Kiiski et al., 2022). Furthermore, it is possible differences in theta activity and the theta-beta ratio can also occur in other disorders, such as epilepsy, dementia, alcoholism, and schizophrenia, and is not specific to ADHD or useful for its distinction (Newson and Thiagarajan, 2019).

### 1.3 Current study

First, we expected the level of electrical activity in the brain would generally decrease (i.e., in average activity of all participants) from the eyes-closed to eyes-open condition as has been observed in previous studies (e.g., Barry et al., 2007; Barry and De Blasio, 2017). Second, we hypothesized that theta and delta frequencies will be increased in the ADHD group compared to the control group replicating the results of Kiiski et al. (2022) and the collective findings reviewed by Newson and Thiagarajan (2019) in parietal-occipital regions and overall. Third, based on the existing evidence that beta bands are linked to sensorimotor activation (e.g., De Castelnau et al., 2008), we expect increased power in frontal and central beta frequencies will be present indicating impairment among those with DCD compared to typically developing adults. Considering the findings for alpha frequencies are mixed, we will also examine if in frontal and central alpha are increased in DCD in line with de Castelnau et al. (2008), or if there are no between-group differences in alpha power in line with Keating et al. (2023). As there are no existing studies to indicate the general frequency band patterns in participants with DCD + ADHD or to examine differences at rest between DCD and ADHD groups, we exploratorily compare frequency band activity between all groups (i.e., DCD, ADHD, DCD + ADHD, typically developing) by brain region. Exploratory correlation analyses will also be conducted to compare symptom severity to spectral power.

## 2 Methods

### 2.1 Participants

A total of *N* = 46 adults were included in the present study. Among them, *n* = 12 had a diagnosis of DCD, *n* = 9 had a diagnosis of ADHD, *n* = 8 were diagnosed with both DCD and ADHD (DCD + ADHD), while *n* = 17 were typically developing, with no known mental or physical health conditions. Participants identified as women (*n* = 35), men (*n* = 10), and transgender (*n* = 1). In addition, a majority were right-handed (*n* = 37). They were, on average, 25.8 years old (*SD* = 7.85; Range: 19–53). As adults with DCD in particular can be difficult to recruit, combining test locations is a common approach in DCD research to gather larger sample sizes while the condition remains under-recognized (e.g., Meachon et al., 2021; Miller et al., 2021). Therefore, participants were tested in Germany (*n* = 26) and the UK (*n* = 20). Aside from the language in which the study session was conducted, demographic factors (i.e., age, gender, handedness) neither differed between study groups nor based on test site (see Supplementary material).

Several participants in the clinical groups had co-occurring mental health conditions, including autism spectrum disorder (*n* = 4), dyslexia (*n* = 2), learning difficulties (*n* = 2), and anxiety or depression (*n* = 2). All participants with ADHD were asked not to take ADHD medication for 24 h before the testing session. The study was approved by the local ethics committees at both sites.

### 2.2 Screening and group classification

All participants were screened in line with the DSM-5 diagnosis and current gold standard assessment for adults with DCD (American Psychiatric Association, 2013; Blank et al., 2019). Criterion A for DCD (indicating motor skill acquisition and executive are below peers; American Psychiatric Association, 2013) was confirmed in participants recruited in the UK using the Movement Assessment Battery for Children version 2 (MABC-2; Henderson et al., 2007; see Table 1). In addition, criterion B and C (B: motor skills interfere with daily life in several domains, C: symptoms began in childhood; American Psychiatric Association, 2013) were addressed with the Adult DCD/Dyspraxia Checklist (Kirby et al., 2010; American Psychiatric Association, 2013). Furthermore, presence of ADHD symptoms was assessed with the Adult Self-Report Scale for ADHD v.1 (Kessler et al., 2005; see Table 1).

**Table 1:** Group classification and testing location comparisons.

Groups: overall (*N* = 46)	Sample size (*n*)	Average ADC score (*SD*)	Average ASRS v.1 score (*SD*)	Median MABC-2 percentile
DCD	12	108.2 (22.5)	41.5 (8.8)	N/A
ADHD	9	89.1 (14.5)	59.3 (8.8)	N/A
DCD + ADHD	8	112.0 (18.8)	59.0 (13.4)	N/A
Control	17	69.8 (13.8)	44.4 (11.4)	N/A

Participants from Germany (*n* = 26)	Sample size	Average ADC score	Average ASRS v.1 score	Median MABC-2 Percentile
DCD	2	85.5 (47.4)	43.0 (7.1)	N/A
ADHD	6	91 (15.4)	61.3 (10.5)	N/A
DCD + ADHD	3	118.7 (24.9)	69.0 (8.7)	N/A
Control	15	72.0 (13.1)	47.8 (6.5)	N/A

Participants from UK (*n* = 20)	Sample size	Average ADC score	Average ASRS v.1 score	Median MABC-2 percentile
DCD	10	113.9 (14.9)	41.2 (9.5)	3rd
ADHD	3	85.3 (14.5)	55.3 (0.6)	25th
DCD + ADHD	5	108.0 (16.0)	53.5 (12.5)	5^th^
Control	2	53.5 (5.0)	19 (4.2)	55^th^

Overall group scores on the ADC and ASRS v.1 were compared via a one-way ANOVA. There was a significant effect of group on ADC score [*F*(3, 42) = 16.61, *p* < 0.001]. Tukey’s *post hoc* test revealed group comparisons between the control group versus the DCD and DCD + ADHD groups were significant (*p* < 0.001). There was also a significant effect of group on ASRS v.1 scores [*F*(3, 42) = 8.13, p < 0.001]. Tukey’s *post hoc* test revealed significance (*p* < 0.01) in all group comparisons except for the DCD versus typically developing groups and the ADHD versus DCD + ADHD groups. ADC scores were based on a scale with responses scored from values 1 to 4 as opposed to 0–3. Therefore, cutoff scores are higher than recommendations for >/= 65, instead at 98 and over. ADC, adult DCD/dyspraxia checklist; ASRS, adult self-report scale for ADHD; MABC-2, movement assessment battery for children version 2.

**Figure 1:**
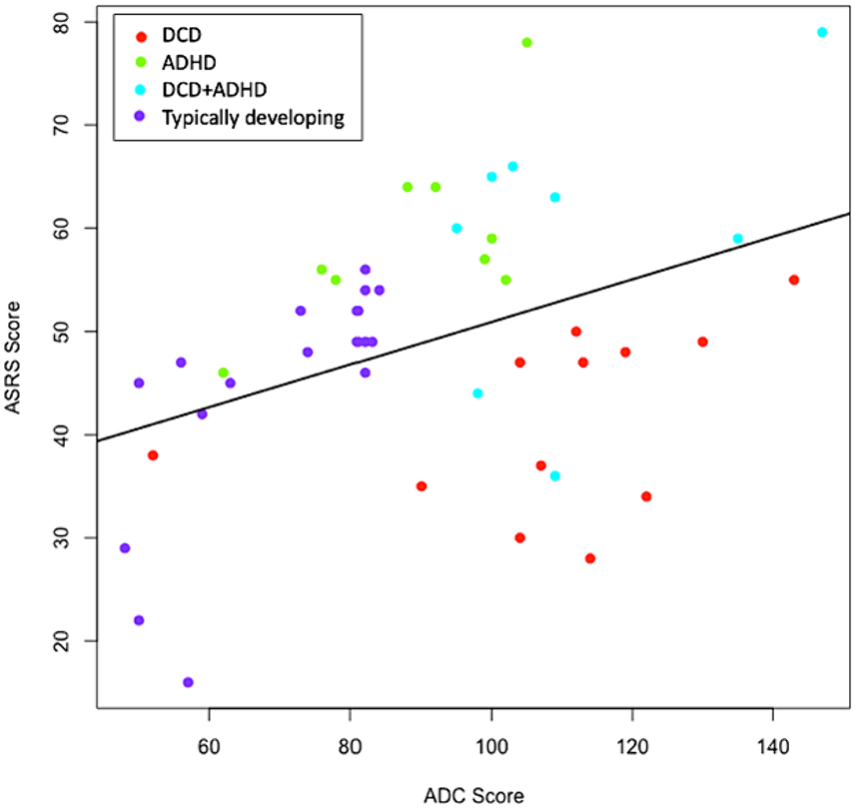
Distribution of ADC x ASRS Scores. Groups were determined by previous diagnosis and confirmed with ADC and ASRS scores. Where discrepancies were present, diagnostic history was favored. The correlation between ADC and ASRS scores was significant (*p* = 0.007) and positive (*r* = 0.395). ADC, adult DCD/dyspraxia checklist; ASRS, adult self-report scale for ADHD.

Groups were classified based on previous diagnosis and confirmed to differ in expected directions based on self-reported symptoms of DCD and/or ADHD (see Table 1). As visualized in Figure 1, some clear group distinctions can also be observed. In some cases, participants had borderline values in self-reported DCD and ADHD symptoms (see Supplementary material), however, they are highly consistent by study group based on previous diagnosis (see Figure 1).

### 2.3 Procedure

Resting state trials included 2 min with eyes-open, and 2 min with eyes-closed, respectively. For eyes-open trials, participants were told to relax and look at a fixation cross in the middle of the screen while preventing head or eye movements as much as possible. For eyes-closed trials, participants were instructed to remain relaxed and awake but still. Participants had the opportunity to take a break and move between eyes-open and eyes-closed trials. The data is a subset of participants who completed resting tasks in the middle of a broader pilot study which included a detailed questionnaire and executive functioning tasks [see Meachon et al. (2021)].

### 2.4 EEG measurement

The EEG measurement took place in a soundproof booth with absence of phones or other technology aside from the study equipment. Participants were seated in a comfortable chair at a viewing distance of approximately 150 cm to the screen, with a visual angle of about 15 degrees, and had a keyboard in front of them to advance between trials during the break via a mouse click. Measurement of the rest state task lasted 4 min total, plus an open-ended break between eyes-open and eyes-closed trials. A black fixation cross was presented in the middle of a gray screen (visual angle: 43° in Germany and 67° in UK). The EEG systems at both sites had 64 electrodes which followed the international 10–20 system (Brain Products GmbH) with a ground electrode at FpCz and Reference at FCz. The impedance of the electrodes was monitored closely as to not exceed 15 kΩ.

### 2.5 EEG pre-processing

Electroencephalography data were recorded at or adjusted to a sampling rate of 500 Hz for consistency between tests sites. All data used band pass filters of 0.5 and 50 Hz in line with similar resting state EEG studies (De Castelnau et al., 2008; Van Dongen-Boomsma et al., 2010; Woltering et al., 2012; Liechti et al., 2013; Buyck and Wiersema, 2014; Alba et al., 2016; Rommel et al., 2017). The reference was computed using the average of all electrodes. When individual electrodes were substantially noisy and/or lack of signal reception was suspected, a topographical interpolation was performed. This was computed in *n* = 5 participants for an average of 1.4 electrodes each. An independent component analysis (ICA) was conducted for each subject to detect and remove eyeblink and movement artifacts where relevant. Finally, artifact rejection was performed to automatically remove noisy epochs for both trial types, leading to a removal of small amounts of data in *n* = 17 participants. The range of artifact removal was from 0.4 s (i.e., 0.003% of the data in one condition for one participant) to 124 s (i.e., 1.1% of the data in one condition for one participant) with a median of 2.1 s. The eyes-open and eyes-closed conditions were pre-processed separately, and each divided into 2 s epochs without overlap. All pre-processing was conducted with Brain Vision Analyzer 2.0 (Brain Products, Germany).

### 2.6 EEG analysis

At each electrode and using a windowing approach, amplitudes were measured for alpha (7.5–12.5 Hz), beta (13–25 Hz), theta (3.5–7 Hz), and delta (0.5–3 Hz), bands in line with existing resting state studies measuring resting states in participants with ADHD (Van Dongen-Boomsma et al., 2010; Woltering et al., 2012; Liechti et al., 2013; Buyck and Wiersema, 2014; Alba et al., 2016; Rommel et al., 2017). We also included two ranges of gamma bands from 30 Hz to 40 Hz, reported in this paper as “low gamma,” and 40–50 Hz reported in this paper as “high gamma.” The mu band was also estimated from 8 Hz to 12 Hz based on existing studies about the motor system (e.g., Perry et al., 2011). We note that the mu and alpha ranges are nearly equivalent, and may be better denoted by region rather than frequency.

Spectral power was computed at all frequency bands in the frontal (Fp1, Fp2, F1, F2, F3, F4, F5, F6, F7, F8, Fz, AF3, AF4, AF7, AF8, FC1, FC2, FC3, FC4, FC5, FC6, FT7, and FT8), centroparietal (C1, C2, C3, C4, C5, C6, CPz, CP1, CP2, CP3, CP4, CP5, CP6, P1, P2, P3, P4, P5, P6, P7, and P8), and occipital (Oz, O1, O2, POz, PO3, PO4, PO7, and PO8) regions during 2 min for each of the eyes-closed and eyes-open conditions. Whole-brain analyses are defined as frequency bands which included all valid electrodes across the scalp, as opposed to specific regions (i.e., frontal, centroparietal, and occipital). Due to a higher degree of noise in some temporal electrodes, as well as some differences in measurement systems between sites (e.g., Iz only used in UK EEG), a temporal region was not assessed. For a cautious approach, these electrodes (electrodes FCz, AFz, Fpz, FT9, FT10, PO9, PO10, P9, P10, TP9, TP10, and Iz) were also removed from the analyses of overall brain activity. In addition, sequences with electric potentials above 100 mV were rejected during data processing. A frequency extraction was performed for each frequency band by specifying windows at the following frequencies: delta: 0.5–3 Hz; theta: 3.5–7 Hz; alpha: 7.5–12.5 Hz; beta: 13–25 Hz; mu: 8–13 Hz; low gamma: 30–40 Hz; high gamma: 40–50 Hz. Arithmetic means were computed for resulting absolute spectral power values during eyes-open and eyes-closed conditions. For a full picture of activity in this exploratory study, this was considered for the whole brain, as well as frontal, centroparietal, and occipital regions. Data was then extracted from Brain Vision Analyzer for statistical comparison.

### 2.7 Statistical analysis

Removal of outliers was performed liberally as to not exclude potential meaningful clinical differences. Therefore, values were removed when they were above three standard deviations from the mean and cross-checked with Q–Q plots. This resulted in the removal of 9 values across all participants for all spectral power averages in the eyes-open condition, and 27 values across all participants for all spectral power averages for the eyes-closed condition.

For within-subject comparisons between eyes-open and eyes-closed conditions, a paired-samples *t*-test was conducted. Given that there are just two conditions (*k* = 2), a Bonferroni correction was not needed [i.e., at 5% significance: 0.05/*c;* where *c* = *k*(*k*-1)/2, is equal to significance *p* < 0.05]. Group differences in average frequency band activity were calculated with one-way ANOVAs and to determine the more specific group differences, Tukey’s *post hoc* test is reported to account for multiple comparisons. Levene’s test of unequal variance was performed to account for small group sizes in the nature of the present pilot study. When unequal variances were found, Welch statistic corrections are reported and the Games-Howell *post hoc* tests, which do not assume unique variances, were used.

Given the possibility for intracranial variance (i.e., Hagemann et al., 2008) or other potential confounding factors, we have also included difference scores between eyes-open and eyes-closed conditions (see Supplementary material).

Associations between symptom severity and spectral power were conducted with Pearson correlations. Analyses were conducted in IBM SPSS Statistics, Version 26.0.

## 3 Results

### 3.1 Eyes-open versus eyes-closed

By majority, there was a significant increase in spectral power for all frequency bands and in overall activity from the eyes-open condition to the eyes-closed condition (see Table 2).

**Table 2:** Frequency band activity compared between conditions of eyes-open and eyes-closed.

Frequency band	Eyes-open *M (SD)*	Eyes-closed *M (SD)*	*N*	Significance value (*p*)
Alpha	12.38 (9.96)	27.8 (25.14)	39	<0.001
Beta	6.18 (3.00)	8.11 (4.14)	40	<0.001
Delta	20.70 (16.25)	27.14 (17.69)	41	0.002
Theta	10.64 (6.40)	14.44 (8.73)	39	<0.001
Gamma low	4.83 (2.63)	6.60 (3.82)	40	<0.001
Gamma high	5.18 (2.70)	6.01 (3.28)	41	0.011
Mu	12.20 (9.85)	26.74 (23.95)	41	<0.001
Overall	10.49 (7.83)	19.97 (13.59)	40	<0.001

Activity across all participants is reported. Gamma low refers to signals between 30 and 40 Hz, gamma high includes 40–50 Hz.

### 3.2 Whole-brain spectral power

During the eyes-open condition, alpha and mu frequency bands reached only marginal significance by group [alpha: *F*(3, 38) = 2.83, *p* = 0.052, η^2^ = 0.182; Mu: F(3, 38) = 2.68, *p* = 0.060, η^2^ = 0.175]. Tukey’s *post hoc* test revealed the difference in alpha activity was driven by significantly (*p* = 0.047) higher values in the DCD group (*M* = 20.55, *SD* = 15.82) than the ADHD group (*M* = 6.50, *SD* = 3.82). There were no significant *post hoc* comparisons for the Mu frequency band. Furthermore, there were no significant differences between groups for alpha, beta, theta, delta, low gamma (30–40 Hz), high gamma (40–50 Hz), mu, and overall brain activation during eyes-open and eyes-closed conditions.

### 3.3 Frontal cortex

There was a marginally significant group difference for beta activity in the frontal cortex in the eyes-closed condition [F_Welch_(3,15.6) = 3.11, *p* = 0.057]. The Games-Howell *post hoc* test revealed this effect was driven by a difference (*p* = 0.043) between the DCD (*M* = 14.52, *SD* = 9.11) and typically developing groups (*M* = 5.64, *SD* = 4.19). All other frequency bands across eyes-open and eyes-closed conditions were not significantly different between groups.

### 3.4 Centroparietal cortex

There were no significant between-group differences for the eyes-open or eyes-closed conditions across all spectral power bands in the central region.

### 3.5 Occipital cortex

Between-group differences were present in occipital electrodes for beta [F_Welch_(3,16.5) = 6.86, *p* = 0.003] and marginally significant for high gamma [F_Welch_ (3,18.3) = 3.01, *p* = 0.057] spectral power for the eyes-open condition. Differences in beta power were primarily driven by the comparison (*p* = 0.001) between the DCD (*M* = 4.04, *SD* = 1.69) and typically developing groups (*M* = 8.64, *SD* = 3.62). For high gamma power, Games-Howell *post hoc* tests showed no group differences. There were no significant group differences in spectral power in the occipital cortex during the eyes-closed condition.

### 3.6 Symptom severity and spectral power

Several correlations were present between severity of DCD symptoms, via ADC scores, or ADHD symptoms, via ASRS scores across all participants. However, all correlations were non-significant when *p*-value adjustments for multiple comparisons were applied. Furthermore, the correlations were not significant when examined with Spearman correlations as a follow-up procedure (see Supplementary material).

## 4 Discussion

Overall, the present study preliminarily demonstrated that individuals with DCD and/or ADHD as well as typically developing adults may exhibit only a few noteworthy differences or trends in neural activity that may relate to symptoms of one or both conditions. When considering differences by task condition, we confirmed there is a generally consistent pattern of increased spectral power from eyes-open to eyes-closed comparisons in our sample. These findings are in line with previous studies of typically developing individuals (e.g., Barry et al., 2007). This result primarily reflects validity of the data and neurophysiological activity during each condition. Furthermore, significant group differences were present in the occipital region (eyes-open: beta) while only marginally significant differences were present in whole brain activity (eyes-open: alpha, mu), frontal beta with eyes-closed, and occipital high gamma with eyes-open. Some of these differences were driven by specific group comparisons which could reflect baseline differences at rest.

### 4.1 Increased activity from eyes-open to eyes-closed

We confirmed most frequency bands were consistent with our first expectation that spectral power frequencies would increase from eyes-open to eyes-closed conditions. These findings are in line with some previous studies of typically developing adults (e.g., Barry et al., 2007; Barry and De Blasio, 2017).

### 4.2 Group differences in resting state activity

There were several noteworthy group differences in spectral power which could relate to unique features of DCD and/or ADHD. Given the preliminary nature of this study, we discuss potential explanations for both significant and marginally significant findings. While we cannot claim marginally significant findings are true or robust effects, we urge future research to consider their potential and retest these findings to reveal if these are trends toward or away from statistical significance.

While we could not confirm our hypothesis regarding increased theta and delta frequencies in the ADHD group, an interesting trend was present for alpha power. The marginally significant difference in whole brain alpha power during the eyes-closed condition was driven by the DCD versus ADHD group comparison, indicating a potential for overall alpha power to distinguish DCD and ADHD. The role of alpha power is generally dominant and often reduced in individuals with ADHD compared to typically developing participants (Barry and Clarke, 2013; Deiber et al., 2020; Debnath et al., 2021), but we could not replicate this pattern in the present study. Given that alpha power comparisons between adults with and without ADHD have been linked to both hypoactivation and hyperactivation, it is challenging to interpret this result (Deiber et al., 2020). Nonetheless, the relevance of alpha spectral power should be continuously explored in adults with ADHD, DCD, and for its potential as a marker to differentiate DCD and ADHD.

Next, we expected that fronto-central beta activity would significantly differ for the DCD and typically developing groups in particular and aimed to explore the alpha activity given that previous results are few and conflicting (De Castelnau et al., 2008; Keating et al., 2023). Alpha activity only differed marginally and for the DCD versus ADHD group comparison. Therefore, our results do not support that there is a difference in alpha power at rest between those with DCD and typical developing adults, in line with patterns also observed in children (Keating et al., 2023). However, alpha could be relevant in the context of distinguishing DCD and ADHD and should be tested further to determine the possibility.

Furthermore, we found a marginally significant group difference in whole-brain mu activity. Given that (a) no group differences could be found via *post hoc* tests and (b) mu waveforms overlap with alpha and can be challenging to disentangle (Garakh et al., 2020), these results should be interpreted with caution. Future research should examine the mu and alpha waveforms and their potential for regionally specific roles in DCD and/or ADHD.

In addition, we found group differences in line with our expectations such that occipital beta power was significantly increased in DCD compared to typically developing participants but this pattern was only observed at marginal significance for frontal beta power. The latter trend is in line with relevance of frontal beta to DCD noted by de Castelnau et al. (2008). Notably, the previous associations between some cases of ADHD and greater frontal beta rhythms (Kropotov, 2016), are also not identified in the present study. Beta waves broadly reflect a wakeful state with mental activity taking place but can also be related to motor initiation and termination as well as motor planning and inhibition (Kropotov, 2016; Heinrichs-Graham et al., 2017; Barone and Rossiter, 2021).

While the differences in beta found in this study should be interpreted with caution and tested further, there are several explanations we theorize might be linked to beta differences in DCD that should be tested further in future research. In general, beta rhythms have been noted to increase after movement, potentially as a result of the motor system regaining balance, adaptation, or regulation (Heinrichs-Graham et al., 2017). Among typically developing individuals, increases in post-movement beta activity were greater when movement was stopped suddenly compared to slowly (Heinrichs-Graham et al., 2017). This pattern should also be tested in individuals with DCD. In addition, substantial increases or decreases in post-movement beta activity can reflect motor learning taking place (Barone and Rossiter, 2021). Therefore, it is possible the elevated beta level in DCD could reflect some degree of novelty of the resting state task specific to this group or a unique motor modulation in line with motor difficulties known to coincide with DCD. Furthermore, the beta frequency may reflect the presence or absence of maintaining one’s cognitive or sensorimotor state (Engel and Fries, 2010). This could indicate a potential difficulty in the transition from movement to rest or maintenance of rest in the DCD group and required less effort from the typically developing group, who likely find it more natural to sit still or fluidly control their posture than those with DCD (e.g., Geuze, 2005; Miller et al., 2019). Thus, resting state activity may originate in structural differences, functional differences of neural networks or different (cognitive) activity during quiet sitting [also see Wilhelm et al. (2001)].

In our study, it is possible that the chairs at different testing locations could support participants in balancing to different degrees, given that more participants in the DCD group were tested in the UK and more of the typically developing group in Germany. However, both explanations would be supported by a consistent beta difference in the DCD + ADHD group (primarily tested in UK), which was not found in this study. As there are several plausible explanations for the observed increases in beta power in DCD at rest which can only be speculated upon in the present paper, future studies should test various contexts of rest and activity in DCD to support determining whether beta could be a potential biomarker for DCD.

Finally, when considering occipital activity, a significant difference in beta in the eyes-open condition was driven by comparisons between the DCD and typically developing groups, and a marginally significant difference was found in high gamma activity with eyes-open. The differences between the DCD and typically developing groups could potentially reflect a difference in visual attention related to the widely known role of the occipital cortex (Gola et al., 2013). Beta and gamma power are often indicative of wakeful and mentally active states, potentially related to higher order cognitive processing (Başar, 2013; Mably and Colgin, 2018). It is possible that participants with DCD needed to modulate their motor activity in posture and to sit still. This might have resulted in a unique recruitment of occipital beta and gamma power, potentially linked to increased effort and/or attention (Gola et al., 2013).

Overall, there are far more similarities in resting state electrical activity than there are significant differences in the present study. It is possible that differences between DCD and ADHD as well as DCD and typically developing participants observed at the external, behavioral, or subjective levels are often subtle in neurophysiology, especially in adults. By adulthood, symptoms of DCD and ADHD could have already been managed in treatment or compensated for on an individual level (Wilmut, 2017). Therefore, it is not surprising that adults with DCD and ADHD have many similarities in the context of a simpler task, but it is all the more remarkable that several key group differences and potential trends were observed in the present study, especially between DCD and typically developing adults.

### 4.3 Limitations and future directions

The present study is considered a pilot study and by nature is under-powered. As studies with comparisons of spectral power DCD and/or ADHD have not been previously conducted, the present study also provides a baseline for effect sizes in future related studies in calculating a minimum sample size and general direction for selecting relevant spectral power bands. Furthermore, within the pilot and exploratory context of the study, we provided conservative corrections and tested across multiple sites to increase the sample size for DCD and DCD + ADHD groups in particular. We considered potential group differences between test sites and while demographics were consistent, it should be noted that more participants with DCD were recruited in the UK sample. Our challenge recruiting individuals with DCD in the German sample is suspected to be due to under-recognition of DCD in German clinicians (Meachon et al., 2023). Nonetheless, we replicated the set-up as closely as possible. Furthermore, some differences between sites and groups recruited are inevitably different. For example, intracranial variance could differ between participants (Hagemann et al., 2008) and coincidentally between groups. Although difference scores did not significantly differ between the groups, it could be assumed the groups had comparable change from eyes-open to eyes-closed conditions (see Supplementary material). However, given the small sample size, replication of the present study is necessary to conclusively determine if resting state differences between DCD and/or ADHD are robust.

Another limitation is the order of the tasks consistently beginning with eyes-open trials and ending with eyes-closed in between measurement of other executive functioning task. While other studies have indicated the increase in power from eyes-open to eyes-closed can be found even when the eyes-closed and eyes-open conditions are repeated many times within one study (e.g., Barry et al., 2007), this should still be considered with a randomized design for future studies on DCD and ADHD. In addition, all participants completed resting state trials in an enclosed room without the experimenter present. Therefore, it is possible some participants moved during the task which could not be detected in EEG artifact analysis alone. While there were no demographic differences based on test site and as many features as possible were kept consistent, it is still possible the different testing locations (e.g., chairs) could have had a minor influence on comfort during the rest task.

Finally, causal links cannot be drawn between specific patterns of electrical brain activity and symptoms of DCD and/or ADHD in this study and should be examined in future research. This could be particularly important in future steps toward determining which endophenotypic features are unique to co-occurring versus single-occurring DCD and ADHD.

## 5 Conclusion

This study provides a foundation for determining the potential for overlap and differentiation of DCD and ADHD through resting state electrical brain activity. Several group differences could be noted in adults with DCD and typical development during seated rest with some potential differences between DCD and ADHD. This suggests that there might be a few fundamental significant baseline differences unique to DCD which are not present in co-occurring DCD + ADHD or ADHD alone. We theorize, but cannot confirm, that resting state behavior can still engage symptoms in DCD, potentially requiring additional motor load to maintain a seated position. Furthermore, numerous overlaps were observed between groups such that spectral power values were not significantly different in the resting state more often than differences were found. Therefore, it is likely the neural mechanisms between DCD and/or ADHD are generally similar at baseline. This is important for the future assessment of DCD, direction of differentiation of DCD and ADHD, and the interpretation of the resting state in general.

## Data availability statement

The datasets presented in this study can be found in online repositories. The names of the repository/repositories and accession number(s) can be found at: https://osf.io/zjws3/ and https://madata.bib.uni-mannheim.de/451/.

## Ethics statement

The studies involving humans were approved by University of Mannheim Ethics Committee; Oxford Brookes University Ethics Committee. The studies were conducted in accordance with the local legislation and institutional requirements. The participants provided their written informed consent to participate in this study.

## Author contributions

EM: Conceptualization, Data curation, Formal analysis, Funding acquisition, Investigation, Methodology, Project administration, Software, Validation, Visualization, Writing – original draft, Writing – review & editing. MK: Data curation, Investigation, Methodology, Software, Writing – original draft, Writing – review & editing. KW: Conceptualization, Investigation, Methodology, Resources, Supervision, Writing – original draft, Writing – review & editing. GA: Conceptualization, Investigation, Methodology, Project administration, Resources, Supervision, Writing – original draft, Writing – review & editing.
