# Supplementary material for: EEG spectral power in developmental coordination disorder and attention-deficit/hyperactivity disorder: a pilot study
Source: Front Psychol. 2024 May 3;15:1330385. doi: 10.3389/fpsyg.2024.1330385 (PMC11099285; doi:10.3389/fpsyg.2024.1330385)
Supplement: Supplementary file 1 [file Data_Sheet_1.docx]

**Supplementary Materials**

Table 1. Demographic comparisons by test site (Germany vs. UK)

| **Demographics** | **Germany** | **UK** | **Sig.**  ***(p-value)*** |
| --- | --- | --- | --- |
| Age: *M (SD)* | 25.46 (8.15) | 26.63 (7.63) | .31 |
| Gender: *M (SD)* | .19 (.40) | .37 (.60) | .14 |
| Female (%) | 81% | 68.4% | -- |
| Male (%) | 19% | 26.3% | -- |
| Transgender (%) | 0% | 5.3% | -- |
| Handedness: *M (SD)* | .81 (40) | .79 (.41) | .44 |
| Right (%) | 81% | 79% | -- |
| Left (%) | 19% | 21% | -- |

*Note:* Group comparisons were examined with an independent-samples t-test. Sig. = significance; gender values were 0 = female, 1 = male, 2 = transgender; handedness: 0 = left, 1 = right

Table 2. Demographic comparisons by group (DCD, ADHD, DCD+ADHD, typically developing)

| **Demographics** | **DCD** | **ADHD** | **DCD+ADHD** | **TD** | **Sig.**  ***(p-value)*** |
| --- | --- | --- | --- | --- | --- |
| Age *M (SD)* | 26.83 (9.81) | 27.67 (6.38) | 26.63 (7.63) | 23.41 (5.96) | .48 |
| Gender *M (SD)* | .25 (.45) | .22 (.44) | .50 (.76) | .39 (.10) | .50 |
| Female (%) | 75% | 78% | 63% | 82% | -- |
| Male (%) | 25% | 22% | 25% | 18% | -- |
| Transgender (%) | 0% | 0% | 12% | 0% | -- |
| Handedness *M (SD)* | .83 (.39) | .89 (.33) | .75 (.46) | .76 (.44) | .86 |
| Right (%) | 83% | 89% | 75% | 76% | -- |
| Left (%) | 17% | 11% | 25% | 24% | -- |

Note: Group comparisons were computed with a one-way ANOVA; TD = typically developing; gender values were 0 = female, 1 = male, 2 = transgender; handedness: 0 = left, 1 = right

Table 3. Differences scores between eyes-open and eyes-closed conditions where significant group differences were found in main analyses

|  | **DCD** | | | **ADHD** | | | **DCD+ADHD** | | | **TD** | | | |
| --- | --- | --- | --- | --- | --- | --- | --- | --- | --- | --- | --- | --- | --- |
| Region, waveform | Eyes-open | Eyes-closed | Difference | Eyes-open | Eyes-closed | Difference | Eyes-open | Eyes-closed | Difference | Eyes-open | Eyes-closed | Difference |  |
| Whole-brain, alpha | 20.55 (15.82), *n* = 12 | 41.33 (35.96), *n* = 10 | 23.53, *n* = 10 | 6.95 (3.82), *n* = 7 | 36.11 (34.73), *n* = 9 | 19.87, *n* = 7 | 13.13 (7.89), *n* = 8 | 20.83 (20.57), *n* = 7 | 9.29, *n* = 7 | 11.70 (8.26), *n* = 15 | 25.28 (18.81), *n* = 17 | 10.80, *n* = 15 | |
| Frontal, beta | 10.27 (6.10), *n* = 12 | 14.52 (9.11), *n* = 11 | 4.45, *n* = 11 | 6.40 (6.89), *n* = 8 | 9.53 (10.69), *n* = 9 | 2.55, *n* = 8 | 9.79 (7.55), *n* = 8 | 8.38 (5.94), *n* = 7 | .50, *n* = 7 | 5.04 (2.61), *n* = 15 | 5.64 (4.19), *n* = 17 | .82, *n* = 15 | |
| Occipital, beta | 4.04 (1.69), *n* = 12 | 6.28 (3.94), *n* = 11 | 2.30, *n* = 11 | 6.28 (3.00), *n* = 8 | 11.96 (7.42), *n* = 9 | 6.25, *n* = 8 | 11.79 (12.96), *n* = 8 | 11.47 (16.42), *n* = 7 | 3.40, *n* = 7 | 8.64 (3.62), *n* = 15 | 14.01 (7.27), *n* = 17 | 4.26, *n* = 15 | |
| Occipital, high gamma | 4.02 (2.09), *n* = 12 | 5.53 (2.88), *n* = 11 | 1.36, *n* = 11 | 3.49 (1.44), *n* = 8 | 6.14 (4.23), *n* = 9 | 2.46, *n* = 8 | 14.20 (13.94), *n* = 8 | 5.75 (5.94), *n* = 6 | 2.55, *n* = 6 | 5.31 (2.28), *n* = 15 | 7.32 (3.76), *n* = 17 | 1.22, *n* = 15 | |

*Note:* Values reported are M (SD). Difference scores are the absolute value of *M*(Eyes-open) – *M*(Eyes-closed). Where values were not available in both conditions, participants were excluded in the difference score calculation. None of the difference scores differed significantly between groups (*p* > .05).

**Group Classification and Borderline Scores**

A total of *n* = 21 participants were above threshold for likely DCD (Kirby et al., 2010), however, *n* = 4 of these participants had ADHD. In addition, there were *n* = 2 participants with DCD and *n* = 1 with DCD+ADHD who did not score above this cutoff. For discrepancies in ADC scores, diagnostic history was preferred for group classification due to the high chance for individuals with ADHD or ASD to also score high on the ADC (Kirby et al., 2010; see Figure 1). Of those tested with the MABC-2, a total of *n* = 15 participants fell below recommended cut-off score (16^th^ percentile or lower) for likely DCD. One participant with DCD scored above the 16^th^ percentile but had a high ADC score and one participant with ADHD scored below the 16^th^ percentile but had an ADC score just above the cutoff score for likely DCD. Therefore, they remained in groups according to their original diagnosis. Notably, the co-occurring conditions reported by participants did not better explain a diagnosis for DCD, addressing criterion D for DSM-5 diagnosis (American Psychiatric Association, 2013). Previous diagnosis of ADHD was required for group assignment to the ADHD and DCD+ADHD groups. As it is possible that individuals with DCD and not ADHD score high on the ASRS due to DCD symptoms alone, previous diagnosis took precedence for group classification regarding ADHD.

**Correlations non-significant after correction for multiplicity**

For DCD symptoms, significant negative associations were found for occipital alpha (*r* = -.335, *p* = .030), delta (*r* = -.319, *p* = .035), theta (*r* = -.310, *p* = .041), and mu (*r* = -.341, *p* = .027) in the eyes-closed condition (see Figure 2). Furthermore, ADHD symptoms correlated significantly and negatively with frontal spectral power for theta (*r* = -.312, *p* = .042), and low gamma power (*r* = -.309, *p* = .044) in the eyes-closed condition, as well as overall activity in all regions for the eyes-closed condition (*r* = -.305, *p* = .047; see Figure 2). DCD and ADHD symptoms themselves were also highly correlated (*r* = .395 *p* = .007). All aforementioned correlations were non-significant when examined with Spearman’s correlation coefficient. Figure 1 depicts this relationship across participants in all four groups. No other correlations across all conditions (i.e., eyes-open), regions, and frequency bands were statistically significant (*p* > .05, non-adjusted).

**Figure 2.** Significant correlations between ADC or ASRS scores x spectral power.


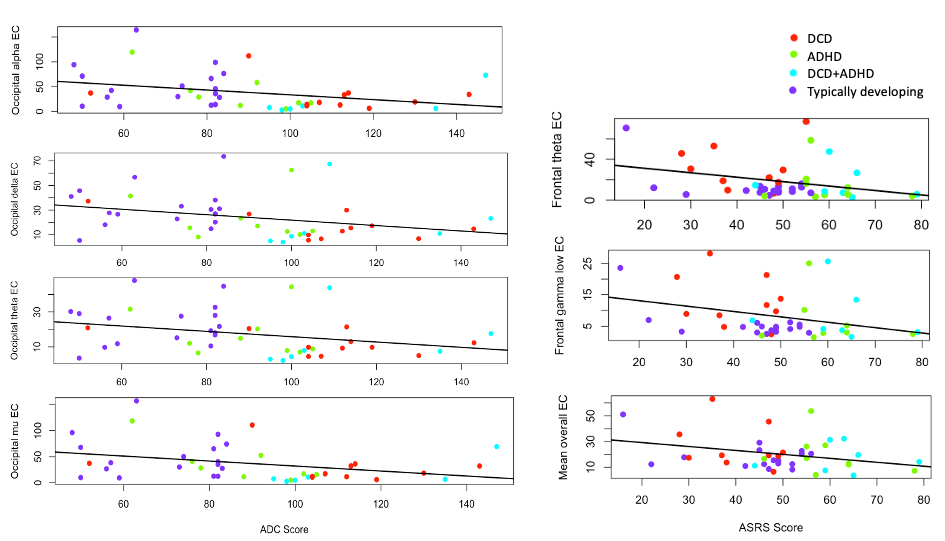

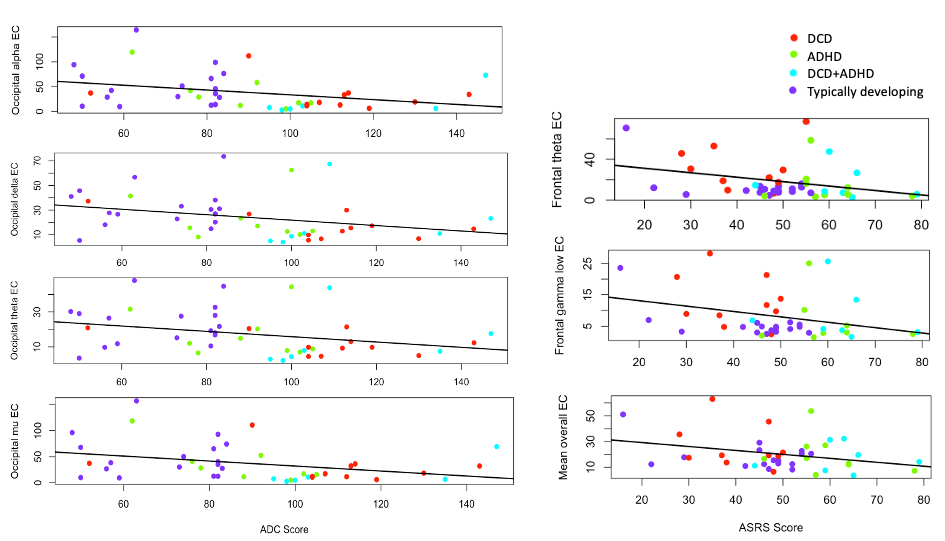


*Note*: The ADC was used to measure motor symptoms and probable DCD. The ASRS was used to measure inattention hyperactivity and probable ADHD. All spectral power values were in the eyes-closed condition, EC = eyes-closed; Mean overall EC = average overall spectral power during the eyes closed condition.
